# Singing lessons as a path to well-being in later life

**DOI:** 10.1177/03057356211030992

**Published:** 2021-09-03

**Authors:** Alexandra M Smith, Kay Kleinerman, Annabel J Cohen

**Affiliations:** 1 Department of Psychology, University of Prince Edward Island, Charlottetown, PE, Canada; 2 Independent Voice Teacher, Oakland, CA, USA

**Keywords:** singing, older adults, well-being, physical health, mental health

## Abstract

Seventy-two persons, who had begun voice lessons after 40 years of age, were invited to complete an online survey that focused on the singers’ experience, motivation, goals, health and well-being, repertoire, practice, and demographic information; 48 respondents (33 females, mean age 60.81 years, range 48.83–82.08, *SD* = 6.99) completed the questionnaire. Most participants indicated that enjoyment and personal growth motivated their taking lessons. Over 90% commented on benefits of singing to their physical health (e.g., breathing) and mental health (e.g., mood, less depressive episodes). Despite the solitary aspect of singing lessons, 67% reported positive changes in social relations since taking lessons. Benefits to professional relations were also reported (e.g., confidence, listening to others). Repertoire level was generally high, consistent with a high average university educational level. Cost of lessons and time demands may account for the generally high socioeconomic status of respondents. Given that the singing voice is a musical instrument available to almost everyone, results might motivate older adults to consider taking voice lessons, encourage health care professionals to consider voice lessons as interventions to benefit clients, and persuade governments to subsidize voice lessons for older adults in their jurisdictions. The study provides a foundation for future research on the relative impacts on well-being of vocal lessons versus choral singing in the context of relative investments in the two activities.

## Singing lessons as a pathway to well-being in later life

I’m standing on London’s Fleet Street outside an imposing door that’s sandwiched between a solicitor’s office and Ye Olde Cock Tavern. I feel nervous and sticky-palmed. A message pings into my phone. It’s from my 17-year-old daughter. It says: “Try not to worry Dad, it’s only an hour and then it’s over forever, and you never need to do it again. Love you!” I swallow hard and ring the bell. There’s no going back. I’m about to have my first ever singing lesson. (Love, 2019)

British journalist Martin Love’s experience might resemble that of other older adults about to take their first singing lesson. Some of them may not even make it to the front step of a voice teacher’s studio for the first lesson. But what happens to those who do? The present article examines the experience of older adults who have taken singing lessons for the first time in later life.

The number of older adults is rapidly increasing due to the aging of the baby boomer generation and ever-improving medicine and technologies. Worldwide, the proportion of adults over the age of 60 is projected to increase from 12% in 2015 to 22% in 2050 (World Health Organization, 2018). Aging is associated with declines in mental and physical capacities, although there is wide variability within any age cohort and across individuals. Declines in health can burden families and communities and increase costs to government. Consequently, interventions that can maintain health and well-being of older adults can have enormous advantages to individuals and society.

Arts-based interventions have been considered as a source of well-being for older adults (e.g., Fancourt, 2017; Noice et al., 2014). Studies comparing older persons with and without music training have shown differences in cognitive capacities favoring musicians. For example, older musicians have enhanced speech-in-noise perception and better auditory working memory capacity (Parbery-Clark et al., 2011) and attention (Zendel & Alain, 2014). Strong and Midden (2020) compared older adult instrumental musicians, half of whom had continued engagement in music and half who were no longer active. Compared to a group of older non-musicians, both musician groups had higher performance on tests of language and executive functioning; however, the still-active musicians performed better than both of the other groups on another (Stroop-like) test of executive functioning. Recent systematic reviews of studies comparing older musicians and non-musicians have also revealed an association between musical training and cognitive reserve. One meta-analysis that included 11 studies published in English and Spanish showed a relation between music practice and performance on cognitive tasks in persons all over the age of 59 years (Román-Caballero et al., 2018), while a scoping review with 11 English language studies of persons aged 50 years or more, reported similar findings (Schneider et al., 2019).

Musical training is often associated with improved performance in tasks involving memory for musically structured sequences (Cuddy et al., 1981; Fujioka et al., 2004). Most of these studies have been conducted on university students; however, a review by Halpern and Bartlett (2002) of studies conducted with older and younger musicians and non-musicians, revealed that tasks, in which knowledge of musical structure could be exploited, showed higher performance in older musicians as compared to older non-musicians. Sometimes there was no difference between older and younger musicians, although typically younger participants performed at the highest level.

Generally, studies that compare musicians and non-musicians consider the ability to play a musical instrument as the criterion for membership in the *musician* category (e.g., Schneider et al., 2019). If playing a musical instrument contributes to cognitive reserve and social well-being, one might ask whether training in singing might offer similar benefits (Cohen, 2019, 2020a). In support of this view is research of Bialystok and De Pape (2009) showing heightened cognitive performance in both young adult musical instrumentalists and trained vocalists, as compared to those without any musical training. Mansens et al. (2017) conducted a cross-sectional study of over 1,000 persons aged 64 years or more as part of the Longitudinal Aging Study Amsterdam. Engagement in making music was positively correlated with performance on several cognitive tasks. In general, results did not differ for those who played a musical instrument versus those who sang, although processing speed was significantly higher for instrumentalists than for singers. The kind of engagement in singing versus playing an instrument, however, was not carefully examined, with respect to private lessons, or whether playing in a group or solo. It might therefore still follow that benefits of individual vocal lessons and lesson-based vocal practice would show similar benefits to private lessons on another musical instrument. On average, one’s own human voice is more accessible to an older adult than is a musical instrument, because not everyone owns a musical instrument. Such practical access to the vocal instrument adds further support for the goal of the present study which explores the experience of vocal training in older adults.

In contrast to the few studies conducted on the cognitive and social benefits associated with individual vocal training, much attention has been directed to the benefits of group singing (Dingle et al., 2019). Singing in a group has been associated with enhanced social bonding (cf. Maury & Rickard, 2020; Pearce et al., 2015), general feeling of well-being (Clift et al., 2010), sense of personal growth (Noice & Noice, 2009), reduced stress response (Gick, 2011), improved hearing (Dubinsky et al., 2019), reduced pain (Balsnes, 2016; Weinstein et al., 2016), reduced loneliness and increased interest in life (Johnson et al., 2018), heightened immune function (Fancourt et al., 2016), and improved cognitive health (Feng et al., 2020). Some of these studies have focused on choirs comprised of older persons (Balsnes, 2016; Clift et al., 2010; Coulton et al., 2015; Dubinsky et al., 2019; Feng et al., 2020; Lamont et al., 2018; Noice & Noice, 2009).

Although complementary, the experience of singing in a choral group differs from that of engaging in individual singing lessons. The private voice teacher is dedicated to helping the student reach a musical goal, while focusing the student’s attention on the sound of their own voice and its connection to the bodily mechanisms that control it (Beynon, 2020; Cohen, 2020a). The relation between teacher and pupil is complex, and in part depends on the match between personalities (Serra-Dawa, 2014). The teacher potentially enables the learner to achieve increasingly higher musical goals, guiding the student’s vocal growth and mastery of healthy vocal development which, in turn, fosters an even, balanced sound across all notes of the singer’s voice range. In contrast, choral singing typically has the goal of blending one’s voice with those of the other choir members. Practice between rehearsals entails learning one’s part, but not necessarily learning to focus on the mechanics of one’s voice, a key target of voice lessons. This is not to say that choir directors do not or cannot serve valued pedagogical roles (Edwards & Martinec, 2020), and choir directors may, themselves, take voice lessons to help their choristers gain mastery over their voices.

Vocal practice engages a complex sensory-motor network (Tsang et al., 2011). Singing entails focused attention to match the auditory image of intended required notes and phrases with a vocalized pitch. The matching process requires co-ordination of motor neurons that control breathing, stretching, and tensing of the vocal cords, and altering the vocal tract (e.g., articulators, tongue, lip, and jaw position; Cohen et al., 2020; Sundberg, 1987). Vocal practice also exploits memory, for example, remembering prior tones as reference points for future tones, memory for tones within phrases, and phrases within larger segments. Thus, vocal instruction and practice, which aim at improvement of beautiful accurately pitched and timed sounds, may offer a cognitive intervention that exercises focused attention and memory as well as sensorimotor coordination. Notably, this is the same cognitive training associated with learning to play a musical instrument. Voice lessons also engage the student in a complex social relationship with a voice teacher who guides the student through an enriching opportunity to experience and understand music as a performer, rather than as an audience member. Teachers provide the scaffolding to enable the student to reach increasingly higher levels of competence (Küpers et al., 2015, 2017). Furthermore, voice lessons might help expand the student’s self-concept as a singer/musician while learning and performing repertoire that previously may not have fit their image of a possible self. Voice lessons can serve as the foundation for new social activities such as experience in a choir or musical theater group, or singing with friends, family, neighbors, and colleagues.

The present research aimed to explore the experience of private singing lessons in persons over the age of 40 who have never previously had private voice lessons. The rationale for the study was the general notion that development continues throughout the lifespan (Creech et al., 2014) and that singing may be one experiential dimension which allows for continued growth and development whether amateur or professional. There is little information about the potential for development of the singing voice of older persons—most vocal education being focused on later adolescence and early adulthood. Several prior studies have shown that voice lessons have been transformative in the lives of adult women (Kleinerman, 2008; Patteson, 2000); however, these studies did not focus on the potential value of voice lessons for middle-aged and older adults who had recently begun voice lessons. Similarly, a study by Grape et al. (2002), showing the effect of a singing lesson on physiological and behavioral measures associated with well-being of amateur vocalists, did not consider the variable of age. The study did show that the moods of amateurs were uplifted by their voice lessons, whereas a comparison group of professional vocalists were not similarly affected given the association of singing with earning their livelihood.

The following study thus aimed to provide knowledge about the experience of taking singing lessons for the first time in later life. It was hypothesized that persons who began singing lessons after the age of 40 would report a variety of benefits (not everyone will derive the same set of benefits), consistent with a theory of psychological growth across the lifespan (Creech et al., 2014). The possibility of such benefits were, in general, associated with increased well-being arising from deeper self-knowledge (“finding one’s voice”), increased appreciation of music, and the aesthetic experience of creating music, amelioration of health problems, distraction from problems, and communication through performance. These benefits have been noted in other research on musical engagement of adults (see, for example, a review by Creech et al., 2020, pp. 124–125). It was proposed that respondents might identify physical, mental/emotional, personal, and professional benefits of voice lessons, and that people with more responsibilities to children would begin lessons later in life. As well, findings that supported our thesis would offer evidence that private voice lessons in later life could potentially contribute to the happiness and health of older persons, and further, such evidence could provide a basis for advocacy for greater access to voice lessons for this demographic.

The study also aimed to fill several gaps in knowledge about the development of singing in older adults. Some studies suggest that singing voices of older people decline rather than are poised for improvement (as reviewed by Rodney & Sataloff, 2020). In addition to testing the hypothesis that engagement in voice lessons in mid- and later life would offer benefits, the study was also exploratory, as the authors had no prior supposition regarding the duration of voice training in which middle-aged and older adults might engage, the age (beyond the lower limit of 40 years) for beginning lessons, the age of vocal students (within the limits of 40 to 100 years), and the level of difficulty and genre of repertoire on which lessons were focused. The entire project was part of a major collaborative research initiative focusing on advancing interdisciplinary research in singing (AIRS; Cohen, 2011, 2020b) and fell under the research sub-theme that focused on the benefits of singing for well-being.

## Method

### Participant recruitment

To explore the impact of voice lessons initiated in middle or older age, the study first located later life singers and determined their interest in sharing their experiences with the researchers. This was accomplished primarily through contact with the regional representatives of the National Association of Teachers of Singing in North America and secondarily through the following short notice placed in a Saturday edition of a Canadian newspaper with a national readership:

Are you between the ages of 40 and 100? Did you discover singing lessons at age 40 or older? Share your experiences with us. For more information, please contact laterlifesinging@gmail.com.

The communications took place in 2010 and led to 72 valid replies (mean age 61.2 years, range 49–83 years). Many of them volunteered information about their reasons for taking lessons and the impact lessons had on their lives. These unsolicited remarks encouraged the researchers to devise an extensive online questionnaire by which more definitive details could be obtained.

### Survey questionnaire

The questionnaire of 41 items was designed to run online with Survey Monkey software (see Online Supplementary Materials A, for the complete questionnaire). Approximately half the questions were open-ended. Respondents had the opportunity to describe details such as their musical background, non-musical background (e.g., family, childhood, adolescence, young adulthood, community, environment, opportunities, education, and work/profession), experiences with voice teacher(s) and what was required in voice lessons, practice opportunities, singing goals and achievements, repertoire being studied including what repertoire was the most difficult that had been mastered, changing commitment to learning the elements of vocal technique, future plans involving singing and singing lessons, and how the experience of singing affected the ability for expression in other areas.

Closed questions, requiring numerical, rating scale, or check-list responses, requested specific information such as number of years of voice lessons, frequency of lessons, frequency and length of practice, frequency of other leisure activities (e.g., concerts, museums, film, TV, and radio), and demographic information (e.g., gender, category of income level/ socioeconomic status, occupation, ethnic background, country of birth, marital status, level of education, number of children). Some questions offered a 7-point rating scale to indicate level of improvement (if any) since beginning lessons, and a 3-point rating scale to indicate increase in voice range and difficulty of repertoire. A series of 7-point rating scales was provided to reflect the importance of specific results of singing lessons: increase in voice range, complexity of the musical repertoire, emotional depth of the music performed, artistic experience, understanding of music through (a) focused study of specific piece and (b) public performance.

Several closed questions also offered the opportunity to provide further detail. These included reasons for taking singing lessons, singing goals (repertoire, choral group/solo, performance, other), learning (about singing, singing lessons, yourself, others, anything else), impact on life from the standpoint of physical health, emotional/mental health, personal relations, professional relations, other; and messages to different audiences (women, men, children, parents, teachers/educators, religious leaders, and others) that they might want to express about their experience of singing lessons.

### Procedure

The 72 participants who had previously indicated their willingness to provide information about their experience of singing lessons were contacted via the e-mail addresses they had provided and were sent an information letter and consent form, which, along with the entire study protocol, had been approved by the University of Prince Edward Island Research Ethics Board. The information letter and consent form, entitled “Impact of voice lessons in later life” (see Online Supplementary Materials B for complete script) commenced as follows:

We understand that you began taking singing lessons for the first time after the age of 40 and that you continued them for at least a year. We are interested in understanding your experiences as a later-life student of singing, and we invite you to participate in a research project that explores singing as an avenue for personal growth and wellness in later life singers.

The backgrounds of the two principal investigators who initiated the project were provided, and the project was described as part of a larger project focusing on singing. It was stated that the survey would take from 20 to 60 min, or longer, depending on how much the respondent chose to write; participants were encouraged to answer questions as fully as they wished. The potential participants were informed that they could prepare their answers offline and copy and paste them into the online questionnaire, or they could simply respond to the questions the first time the question appeared. The questionnaire could be completed in more than one sitting. The consent form outlined what was expected of participants, possible benefits, possible risks or discomforts (of which there were none), compensation (none), and information about confidentiality and anonymity.

### Participants

Of the 72 initial respondents who were approached following receipt of their contact information, 48 persons completed the questionnaire (33 females and 14 males). One person did not identify gender and responded to only a minority of the questions. Of these who did not respond, there were 7 who sent regrets and gave such reasons as lack of time, taking on new responsibilities, becoming ill, having a relative who had become ill, not meeting the criteria after all, lacking the necessary computer technical skills to even send back the consent form, and lacking computer access. One person had a technical problem and lost the data file before submitting; one submitted answers as a separate text file but did not complete the rating scale data and his data were not used.

### Data analysis

From each respondent’s submitted answers, the Survey Monkey software provided a file of the responses to open-ended and closed questions. It also provided for each of the 41 questions a listing of all responses to open-ended questions and a summary of the responses to the closed-end portion. The response to each question for each participant was transferred to an Excel file. The quantitative results were analyzed with basic descriptive statistics, correlational analyses, or analysis of variance, as described in the “Results” section.

For open-ended questions, for the purpose of a thematic qualitative analysis, the content of the responses was coded by the first author as a means of determining both typical and unique responses. She had received an undergraduate degree in vocal performance (including recitals, operatic performance, pedagogy), postgraduate voice lessons and a course in musician’s health, training on most band instruments, as well as piano, violin, and double bass, courses in qualitative research, music psychology and basic psychology, and had dedicated time over 12 weeks to this coding task in consultation with the third author. For some questions, over 20 different codes were devised to capture the content for a question, and a response by one participant might include as many as four different codes. Codes were chosen based on main concepts relevant to the survey question. For example, in response to the question “What prompted you to turn to singing lessons,” one response of 271 words highlighted the following four codes: “Sight reading (wanting to learn/improve); making a larger contribution to a choir, chorus, or other vocal group; the desire to join or recently having joined a choir; got inspiration or encouragement from someone.” Twenty additional codes were mentioned by other participants, such as “to expand repertoire.”

## Results

In general, the respondents took the questionnaire seriously, providing detailed answers to open-ended questions, amounting to over 50,000 words (equivalent to 200 manuscript pages). In the report of the results, quotations have been included that represented typical responses; several were also selected to represent unique responses.

The 48 respondents, who were on average 60.81 years old (range 48.83 to 82.08, *SD* = 6.99), had taken singing lessons for an average of 5.51 years (*SD* = 3.43) and had started lessons at the average age of 54.60 years (*SD* = 7.73). The number of years of singing lessons was (not surprisingly) negatively correlated with the age at which they started singing lessons, *r*(43) = −.45, *p* = .001, simply reflecting that the earlier they had started lessons, the more years of lessons they had taken. The age they started lessons, however, was also highly correlated with their current age, *r*(45) = .82, *p* = .001, indicating the close relationship between their current age and the year they started voice lessons (on average, this was within about 5 years of their then-current age).

Related to the age of starting lessons was the number of children that each given singer had. The mean number of children of the respondents was 1.66 (*SD* = 1.26; see Table 1(a)). The number of children was significantly correlated with the age of starting lessons, *r*(45) = .31, *p* = .018, consistent with the view that increasing numbers of children may require more years dedicated to childcare with the consequent delay of available resources (e.g., time and funds) for personal voice lessons. A negative correlation between the number of children and number of years of lessons approached a conventional level of significance, *r*(45) = − .22, *p* = .08, which is consistent with a delay in age of starting lessons dependent on number of children.

**Table 1.** Summary of Demographic Data.

(a) Number of children—*N* = 47 respondents					
0 children	1 child	2 children	3 children	4 children	6 children
21.3%	23.4%	36.2%	12.8%	4.3%	2.1%
(b) Marital status					
Married	Cohabiting	Divorced	Separated	Widowed	Never married
68.1%	6.4%	10.6%	2.1%	8.5%	4.3%
(c) Education					
Bachelor’s or higher	Master’s degree	Doctoral			
93.6%	31.9%	23.4%			
(d) Income level (6.4% did not respond)					
US$0–20K	US$21K–35K (lower middle)	US$36K–69K (middle)	US$70K–99K (upper middle)	US$100K–250K (upper)	Money is no object
2.1%	6.4%	27.7%	25.5%	27.7%	4.3%
(e) Ethnicity (number of respondents)					
Caucasian/	Canadian/	Russian/East	Chinese	Irish–Celtic/	Jewish
White/Anglo-Saxon or WASP	French Canadian	European/European		German–Irish	
19/8/2	3/2	2/1/1	2	2/1	3
(f) Country of birth—45 respondents					
United States	Canada	UK/Ireland	Hong Kong	Germany	France
34	5	1/1	2	1	1

Regarding frequency of practice, 23.9% reported practicing daily, 63% reported practicing several times per week, and 10.9% reported practicing once weekly. None reported anything less (i.e., monthly, several times per year). The average time spent practicing in a session was 47.22 min (*SD* = 21.44), based on estimates of the respondents who frequently reported a range (e.g., 30–60 min practice duration), in which case the mean was entered into the calculation. Such a time commitment represents a significant portion of the day dedicated to solo vocal practice. It is also noted that more than two-thirds of the participants were married (68.1%) or cohabiting (6.4%; see Table 1 (b)), indicating that voice lessons were integrated into family life, for the majority. It was thus not the case that those taking voice lessons in later life typically lived alone, could practice without others in the household at any time of day, or were without demands of a family.

Regarding educational level, the group in general was highly educated with 93.6% of respondents having a bachelor’s degree or higher; in fact, 23.4% had a doctoral degree (see Table 1 (c)). Most respondents (91.5%) identified their income level as middle class or higher (Table 1 (d)).^1^ Slightly more than half of the respondents described themselves as “Caucasian” (19) or “White” (8). The others were distributed across 7 self-identified ethnic groups (Table 1 (e)). There were 34 respondents who gave the United States as their country of birth, with seven other countries mentioned (Table 1 (f)). Participants indicated their wide geographical distribution across North America, currently residing in 11 states of the United States and 3 or 4 provinces of Canada (one respondent from Canada did not provide the name of the province). One person indicated residing in the United Kingdom.

### Reasons for taking singing lessons

**Figure 1.**
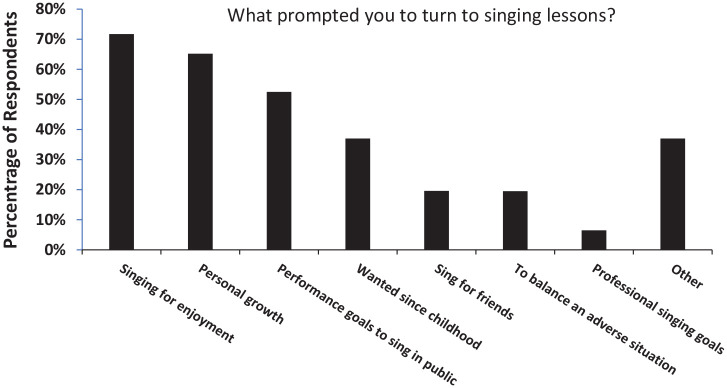
Mean Proportion of Respondents for Whom a Particular Reason Prompted Beginning of Singing Lessons, Checking All That Applied.

Participants had been asked to select any of eight different given reasons for taking singing lessons. Figure 1 shows the percentage of respondents prompted by each of the reasons, with the opportunity to check as many as were applicable to them. On average, the respondents checked more than one option (typically 3). The most popular reasons were “singing for enjoyment” (71.7%), and “personal growth” (65.2%). For 37%, singing lessons now fulfilled a childhood dream; they “wanted to since childhood,” while 37% indicated there were “Other” reasons, for which there was opportunity to elaborate in the open-ended section associated with this question.

Indeed, 89.1% (41) of respondents elaborated with a written response. The most common reason given here for taking singing lessons was that they had joined or wanted to join a choir (13 of the 41 respondents to the open-ended question). The next most common reason given by 7 persons was that they had received encouragement or inspiration from someone (e.g., family member, choral director). Wanting to increase their confidence about their voice was the reason given by 4 persons. There were 3 respondents who wanted to turn to the voice because of time off from playing an instrument (in 2 cases caused by an injury); 3 spoke of turning to singing lessons due to an emotional crisis (e.g., death of a spouse). Other reasons were to help with/prevent vocal health issues. Unique responses included wanting to know more about the physiology of singing, wanting to work in the music field, wanting to improve skills as a church cantor, and wanting an outside opinion of their voice.

### Specific singing goals

Regarding specific singing goals, four choices had been provided: repertoire, choral or solo singing, performance, or other. All three of the goals were regarded as important by the majority (65.9%, 77.3%, and 75%, respectively), while 43.2% indicated there were yet other singing goals. There were 42 individual written responses that elaborated further. For example, there were nine expressions of wanting to improve confidence while singing, and an equal number wanting to improve technique, and two wanting to continue to progress while avoiding the recurrence of vocal nodules or pain.

### What was learned in the lessons

Regarding what was learned in lessons, 100% of the respondents not surprisingly indicated that they had learned about singing and singing lessons, but more importantly, 93.3% indicated learning about themselves, and 71.1% indicated learning about others. In addition, 40% indicated learning beyond these categories. Unique responses included learning the joy music brings them, appreciating their voice teacher, and that people who can sing should use their gift. There were 14 persons who claimed being unaware of technical aspects of singing when they began lessons; the majority (29) however, were committed to the importance of studying technique from the beginning. One person noted only becoming committed to lessons after noticing improvements in their singing voice.

### Teachers and requirements of lessons

Further insight into the experience of lessons arises from answers to the question “describe your experience with your teacher(s) and what was required of you in your voice lessons.” Most respondents spoke favorably about their relationship with their teacher, respecting their expertise, sensitivity to their needs, constructive criticism, expecting the best, expecting weekly practice. At one extreme is the student who expresses:

I love my voice teacher. We do a lot of exercises: breathing, vocalizing, and then we work on various pieces. My range is so much more, now. I can not believe it. It is a lot of work, but so much fun.

This sentiment is echoed in the following comment:

My current teacher is wonderful. She is so positive and uplifting—I like to think of it like some people go get a massage—it is a total yoga and relaxation. . . . What I love about my teacher is that she critiques without criticizing, and she never makes a fuss about how much I practice (or don’t’).

Several other examples serve to reinforce this synergy between adult student and teacher: “We immediately hit it off and became friends which helped greatly with my tension issues.” “I have an excellent voice teacher who is always seeking different ways to help me improve my singing.”

My teacher is fantastic, and I feel very fortunate to have had such a knowledgeable and supportive instructor in my life. I am sure you have heard of teaches the “whole child.” Well, this suites [*sic*] the way my vocal instructor approaches her students.

My second and current teacher these past 5 years is a jewel . . . My lessons are joyous—my teacher is patient, supportive, encouraging. We work hard [he says I work as any of his students]. Music is fun.

Not all relations with teachers work out well, and several of the respondents describe leaving a teacher, for example, who dwells on his own professional experiences rather than focuses on the student. “I never quite found the words or the right moment to ask him to please stop wasting precious, expensive lesson time.” The profound feeling of finding the right teacher is expressed in the following:

When I found my current voice teacher, through a referral, I knew I’d come home. There was an instant connection, and, together, we’ve worked through lots of emotional roadblocks. I now feel like I’m a REAL SINGER and I’m even learning to sight sing.

Disillusionment is the tone of a student who received conflicting information from successive teachers: “Each teacher had something different to bring to the table. One would say mouth not too open, another would say mouth open huge—I found that confusing.”

The importance of enabling the student to self-correct is appreciated by several students: “My teacher is very positive and yet tells me what I need to correct.”

I have noticed that as I get better, it takes less time to work on a particular song, before we move on to another. Either that, or [xx first name of teacher] trusts that I know enough and have taken enough “notes” to work alone on “finishing” a song up.

Students appreciated being pushed: “He pushed me harder than anyone ever did and was a lot pickier, which I needed.”

In terms of technical aspects of the lesson, most of the respondents refer to beginning the lessons with vocal warm-ups and a focus on breathing. A number mentioned that the teacher requires the recording of lessons, and the students spoke positively about the advantage of these recordings for purposes of practice. Several students commented on their valuing the teacher’s knowledge of the science of singing (e.g., physiological information) and avoiding discussions that seemed to be not based in fact. As one student put it: “He has been excellent for me because he knows enough of the true physiology of singing that he does not talk about sounds coming out of your forehead and other such strange notions.” Sensitivity to the older adult was appreciated by several students, “She adjusts the vocal exercises for the week as needed that complement my health issues” although this does not necessarily apply to older persons. Similarly another student said that her teacher “is very conscious of not pushing to strain my voice and she has wonderful warm ups and methods which enhance breathing, vocal placement. I am required to practice, practice and practice.” Common to these diverse experiences is the sense that the relation between the teacher and student seems key to the motivation and sustaining of lessons. Attributes of the teacher go beyond vocal expertise and entail a complex sensitivity to the needs of the student, and the ability to enable the student to imagine and realize their best selves.

The respondents had been asked at the end of the survey whether their voice teacher was a member of NATS (National Association of Teachers of Singing). Of the 44 respondents, 24 indicated that their voice teacher was a member, 2 indicated that the teacher was not a member, and 18 indicated that they did not know. Because the criteria for full NATS members entail a lower age limit of 25 years, regular weekly teaching, and evidence of relevant study, it is likely that the majority of the teachers were well accredited and highly experienced.

### Dimensions of vocal learning

**Figure 2.**
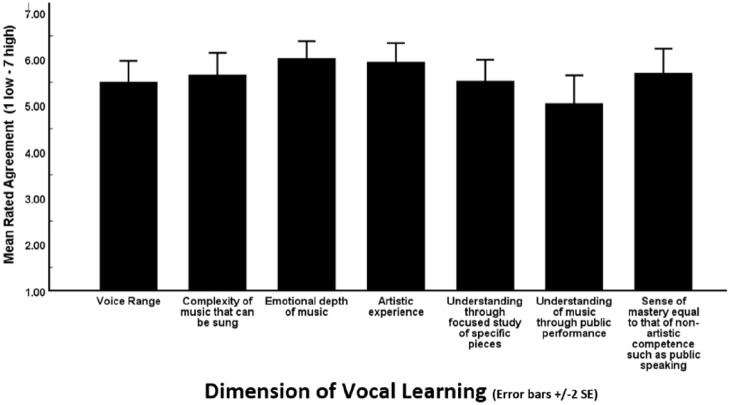
Mean Rated Agreement That Vocal Learning Took Place on Dimensions of Voice Range, Complexity of the Music That Can Be Sung, Emotional Depth of Music, Artistic Experience, Understanding Gained Through Focused Study of Specific Pieces, Understanding of the Music Gained Through Public Performance, and the Sense of Mastery Gained That Was Equal to That of Non-Artistic Competence Such as Public Speaking.

Focusing specifically on what had been learned in regard to singing, participants had been asked to rate the extent to which they agreed that through their lessons they had made progress in terms of voice range, complexity of the music that they could sing, emotional depth of the music, artistic experience of singing, understanding through focus on specific pieces, understanding of music through public performance, and a sense of mastery equal to that of non-artistic competence such as public speaking. See Figure 2 for a plot of the mean rated agreement for each of the seven dimensions of vocal improvement. Overall, the mean rating was high at 5.63 (*SE* = .17, confidence interval [CI] = [5.31, 5.95]). To determine whether it was thought that improvement had occurred on any dimensions in particular, a one-way analysis of variance was carried out on the judgments of the seven dimensions. A Mauchly’s Test of Sphericity indicated the need for adjusted degrees of freedom and, using the Huynh–Feldt adjustment, the main effect of dimension of improvement was statistically significant, *F*(4.50, 206.91) = 2.85, *p* < .02, $\eta_{p}^{2}$ = .06. Pairwise comparisons revealed that the two dimensions receiving the highest ratings (i.e., emotional depth of the music, and artistic experience) were significantly higher than the dimension of understanding of the music through public performance which had received the lowest rating. With the exception of understanding gained through public performance, from a statistical standpoint, participants agree that progress on all remaining six dimensions was equal.

### Impact of singing from the standpoints of physical and mental health, personal, and professional relations

**Figure 3.**
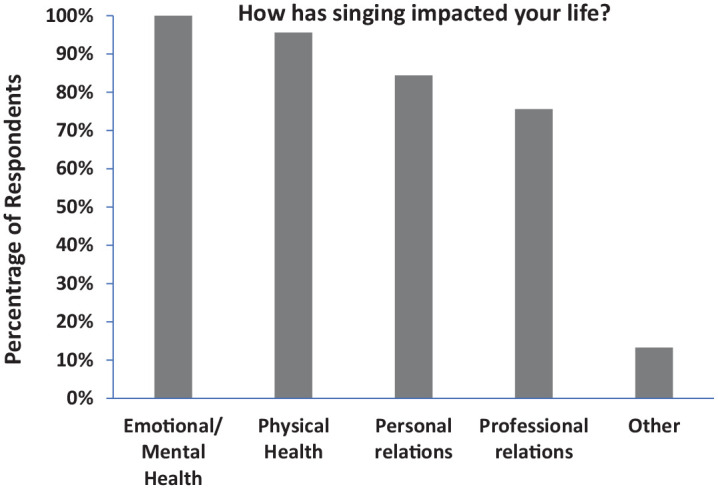
Mean Proportion of Respondents Who Reported Their Well-Being Had Improved on Specific Dimensions, Checking All That Applied.

Participants were asked to indicate whether “singing may otherwise have impacted your life from the standpoint of physical health, emotional/mental health, personal relations, professional relations (e.g., leadership, interaction with colleagues and/or clients) and other. With the exception of the “other” category, over 75% indicated a change to each of these areas, with over 95% indicating an impact on their emotional/mental health and on their physical health (see Figure 3). The open-ended responses provide further insight as described below.

#### Emotional/mental health

There were 45 respondents who commented on benefits to their emotional/mental health, with 17 reporting on the mood-lifting nature of singing as well as the singing lessons themselves, using such terms as feeling “happier,” “lighter in spirit,” and “having less depressive episodes.” One 60-year-old male wrote: “ . . I think I have felt more positive and upbeat. I have joked with my teacher that she is my therapist. And she tells me that her lessons are cheaper than counseling.” Another 59-year-old male put it this way: “Getting lost in singing is a great release, and one of the high points of the week. I’m sure it helps me stay sane.” The second most frequent response (11) was that singing lessons reduced stress “Singing is a stress-reliever for me and helps me combat depression” (49-year-old female), and “Singing is my stress release!” (56-year-old male). There were 6 respondents who reported greater confidence, for example “I have a lot more confidence as a person, not only in my singing, but in knowing there is something I can do well” (70-year-old male).

#### Physical health

There were 43 respondents who described “how singing impacted their life from the standpoint of physical health.” The most common responses were better breathing and increased awareness of their physical health and body. Approximately one-third (15) of the respondents to this question reported that taking singing lessons helped them breathe properly or more fully. For example, a 67-year-old female participant stated that “Singing gives me instant proper breathing feed-back (I have scoliosis and therefore a distorted diaphragm); Utter and deeply relaxing.” There were 8 respondents who reported better physical health/body awareness and 4 reported better posture. For example, one 63-year-old male reported that “It has forced a physical self-awareness—posture, breathing, stamina. My posture has improved.” Several other physical health benefits were reported such as decrease in the number of colds (3 respondents) and less frequent vocal tics (one participant with Tourette’s syndrome). One person reported improved hearing:

I have better air now than twenty years ago when I was a smoker and not singing. I am nearly deaf in one ear. Oddly, I can hear better now than I could four years ago when first I started singing.

Yet five others claimed that they were unaware of any impact on their health, some point out they were already in good health “I don’t think it’s affected my (relatively good) health” (Male, age 55.6). One male (age 60.6) took another stance, noting that his good health contributed to his ability to sing “ . . . I have always been cognizant about how my good health contributes to my ability to sing better, particularly as it relates to breath control”.

#### Personal relations

Of the 38 respondents to the request to “describe how singing may otherwise have impacted your life from the standpoint of personal relations,” 36 reported positive changes in their personal relations since starting voice lessons. Of these, 22 respondents noted socially beneficial aspects of singing lessons, giving reasons such as now having the capability to audition for and participate in choirs and theater productions. Meeting new people who share similar interests has also expanded their social circles immensely. A 60-year-old male said, “personally, as my singing improved and as I got more involved in community theatre, I met a whole new pool of people here, some of whom I consider my closest friends.” Four respondents referred to the support from family of their singing and two reported better relationships with family. For example, a 64-year-old female participant wrote: “My husband encourages my singing because I am more relaxed and easier to get along with!” Personal attributes that helped respondents in relating to others such as confidence and empathy were also reported. A 50-year-old female said, “I become less of an introvert.” Another respondent said, “I think I may be a little more empathetic, perhaps even more extroverted” (60-year-old male, who had taken voice lessons for 6 years).

#### Professional relations

Of the 34 respondents to the request to “describe how singing may otherwise have impacted your life from the standpoint of professional relations,” there were five reports that singing lessons had no impact, and two indicating that the question was not applicable. This was more than in other categories, yet still by far the minority of responses. The majority of respondents did report some benefit, and together the benefits were of wide variety. The most common responses were of increased confidence or self-esteem at work and an increased ability to learn characteristics that were needed to interact well with others in a workplace environment such as confidence and listening skills. For example, a 64-year-old female participant said, “I can practice giving and taking, being in the “lead” (melody) or support—these are good skills.” A 62-year-old male participant noted that “I have become more tolerant of people who don’t get it the first time. Or the second. Or the third.” Such skills may be important for success in the workplace.

#### Other

There were only six responses in the “other” category, and one of these may be representative “I’m running out of things to say . . ” (male, 59.4), with other remarks falling into and reinforcing the previous categories (e.g., “owning my singing voice is the best thing I’ve ever done for myself” [female, age 65.10]) and offering no further insights.

### Repertoire

There were 45 respondents who provided the names of songs they were working on in their lessons. The wide range of pieces could be categorized into 12 genres: Operatic aria, German lieder, art song, jazz, choral/oratorio, French mélodie, early music, traditional, Gilbert and Sullivan, musical theater, pop, and country. There were 15 respondents who indicated pieces of the operatic aria genre, 12 of the art song genre, and 10 of the German Lieder genre. Respondents tended to work on classical songs more than songs in the other categories of pop, musical theater, or country songs.

Repertoire was then compared to the 2019 Royal Conservatory of Music (RCM-Toronto) syllabus to determine average levels according to difficulty of specific pieces. There are 10 RCM levels ending in a final Diploma (11th level). These 11 levels are divided into Elementary (levels preparatory to level 4), Intermediate (levels 5 to 8), Advanced (levels 9 and 10) and Diploma (Associate Diploma in Voice, Performer [ARCT]). The levels are created based on repertoire, theory, and in higher levels, counterpoint and history (The Royal Conservatory, 2019). In terms of a British equivalency to the Canadian system, the Associated Board of the Royal Schools of Music (ABRSM) has a similar grading system, but with fewer levels. The ABRSM refers to their *levels* as *grades*. Grades range from 1 to 8. Upon comparison, grades 7 and 8 of the British system are similar to levels 9 and 10 in the RCM Toronto. RCM Repertoire levels include a variety of lists based on genre or stylistic period, and the performer must choose one song from each list. For example, levels 1 through 6 are encouraged to perform Folk Songs/Pre 1900 Repertoire, 20th/21st century repertoire, and popular repertoire. ARCT candidates must perform Baroque/Classical, Romantic/Post-Romantic, 20th/21st century, Opera Arias, Oratorio/Mass Arias, and Musical Theatre/Operetta (see further the syllabus of The Royal Conservatory, 2019).

Although not every song mentioned by participants could be compared to a level in the 2019 syllabus, most repertoire studied by respondents was at a high level (levels 9 to ARCT). For example, a 60-year-old male was working on *Adelaide* by Ludwig van Beethoven, which is identified in List A (Baroque and Classical Repertoire) at the highest (ARCT) level in the RCM 2019 *Syllabus* (p. 90). A 59-year-old female was working on *Seguidilla murciana*, which is part of a song cycle by Manuel de Falla and is also at the ARCT level (List B, Romantic and Post-Romantic Repertoire, p. 91).

## Discussion

To recapitulate, the study was conducted to fill a gap in knowledge about the experience of taking singing lessons for the first time in later life. Data were collected to test the hypothesis that persons who began singing lessons after the age of 40 would report a variety of benefits consistent with a theory of psychological growth across the lifespan (Creech et al., 2014). The study also aimed to add knowledge about the potential for development of singing skills specifically, and of well-being in later adulthood in general, combatting folk “wisdom” that singing and singing lessons are primarily for the young. The study was exploratory also to the extent that it aimed to obtain information regarding the age at which older individuals might begin lessons, the number of years they would continue with lessons, the level of difficulty and genre of repertoire on which lessons were focused.

### Review of main findings

The positive change reported by 100% of the respondents regarding their emotional and mental health (Figure 3) unequivocally substantiates the potential value of private voice lessons for this age group. This finding alone seems sufficient to add private voice lessons to the list of arts-based interventions for older persons (such as dance, theater, choral groups; instrumental music lessons; e.g., Clift et al., 2010; Lamont et al., 2018; Noice et al., 2014). Benefit to physical health was also a close second to that of the mental health benefits and matches references to improved physical health found in a choral settings (Balsnes, 2016), including improved hearing in one example (Dubinsky et al., 2019). Despite vocal lessons seeming, on the surface, like an isolating activity, respondents reported social and professional benefits, as well as coming to know themselves and others. One might relate this to an idea of possible selves that Creech et al. (2014) have discussed. They speak of the redefinition of one’s musical identity through later life engagement in musical activities, in terms of purpose (associated with structure, new skills and practice), autonomy (associated with freedom, self-expression, confidence), and affirmation/validation (e.g., social affirmation, giving back to the community). These three dimensions, they say, lead to the imagination of possible selves that include such concepts as I am a musician, music is a new opportunity, and I have rediscovered a lost musical self. The experience of voice lessons seems also to have instantiated these dimensions of being a musician (though singing lessons), experiencing the new opportunity provided by music (through singing lessons), and of having brought back (through singing lessons) a skill of singing that was thought of as lost, dormant, or non-existent.

Clearly, the results of the survey show that singing lessons can offer enormous benefits to persons in later life, however, we must ask whether these benefits generalize to older persons beyond those in our sample. In other words, to what extent are the respondents representative of older adults in general? Some demographic data would suggest that a disproportionate number of the respondents belonged to a privileged minority associated with high education and socioeconomic status. According to statistics from the American Council on Education (2017), about 35% of American citizens have a bachelor’s degree or higher. However, for our sample, over 90% of the respondents had a bachelor’s degree, with more than half having master’s or doctoral degrees. The data on income level also suggest a higher-than-average socioeconomic status. Ethnicity was also not representative.

### Limitations

A limitation to this study is that the survey lacks questions regarding negative experiences resulting from participation in singing lessons. Although there was an open-ended question asking respondents for “anything else [they] would like to share regarding their experience with singing lessons” and there was nothing substantially negative noted, further prompting into the area of barriers during lessons, as suggested by a reviewer of the article, may have been appropriate. Furthermore, of the original 72 names of individuals who reported taking singing in later life, only 48 carried out the questionnaire. The sample of participants may have included those for whom singing lessons were the most meaningful, worthy of the time to carry out a questionnaire. Some of those who qualified but did not complete the questionnaire had offered reasons such as lack of ability to use a computer: “I no longer wish to participate. It seems too complicated since I have no knowledge of computers. Sorry,” health issues “I’m sorry, I have been unable to do this. I’m flat on my back with 3 herniated disks and many things are going by the wayside,” “I’m sorry—life has gotten in the way, and I won’t be able to participate after all. I apologize for being unresponsive”; “Sorry, I have been inundated with other projects . . .”; “So sorry, I won’t be able to participate since i [*sic*] took on a big volunteering project last month. I just don’t have time anymore. Many blessings.” Such reasons of health and technical challenges may have disproportionately affected an older demographic, and also contributed to a sample with a non-representative high education level (able to manage the technical challenges of completing the consent form and survey) and having access to better health care and support that allowed not only time for lessons and practice, but the time to focus on and complete the questionnaire. It should be pointed out, however, that no one gave a reason having to do with their experience in voice lessons, positive, neutral, or negative, for not continuing to complete the survey. This would suggest that those who completed were not simply those who felt there were benefits from the lessons. Nevertheless, future studies should prompt for negative aspects of lessons, such as demands on finance and time, satisfaction or disappointment with pace of progress and meeting of expectations.

The data for this study were collected over 10 years ago, at the beginning of a major collaborative research initiative on singing involving over 50 active researchers and their students, and over 30 research projects (www.airsplace.ca). For practical reasons, it was not possible to pursue the analysis of these data until some years after they were collected. Yet, there are precedents for the value of analysis of rich behavioral corpora long after the data are collected. As an example, the Child Language Data Exchange System (CHILDES) includes data on child discourse entered as early as 1984 (MacWhinney & Snow, 1985) and has supported over 6500 publications (Bernstein Ratner & MacWhinney, 2019).^2^ Yet some anachronisms are worthy of consideration. For example, some of the technical problems that prevented some participants from responding would likely be less applicable than in the past, given the increasing access to computers and the Internet. This would facilitate obtaining data from a broader demographic. As another example, the absolute value of currency has changed over the past decade, such that descriptive terms for socioeconomic status (e.g., middle-income level) are more valuable than absolute measures of annual income. Vocal teaching methods may change within a decade. On one hand, new digital technologies for recording and playback offer more pedagogical support. On the other, repertoire, curriculum standards and syllabi may change. Nevertheless, an untapped potential of the older voice has likely been constant over this period. The present study is the first to provide a foundation for further work on the contribution of individual voice lessons in this neglected area at the intersection of the two under-studied fields of singing and musical andragogy (adult learning, see Creech et al., 2020).

Looking specifically at the study design, the survey questions were presented in the same order to all participants, and effects of order of presentation may have influenced the outcome. For example, when participants were invited to describe the impact on their emotional/mental health, their physical health, their personal, and professional life, the participants may have provided a description in the answer to the first item that might have been more applicable to a later item, but chose only to offer the point once. Further, additional information about participants, for example, their work status, might have been gathered to provide explanatory context for some of the answers. For example, a response that there is no application of voice lessons to one’s professional life could arise either if the respondent is not working professionally or if the respondent is working but sees no connection between these two aspects of daily life.

As pointed out the corpus of data collected is extremely rich. In retrospect, this may not be surprising. Singing is about expression and it is for many persons a meaningful experience. It is human nature to share meaning. There was evidence that many respondents wanted to tell their story. As such, only a portion of the data collected have been reviewed in this article. A thematic analysis remains to be completed on the non-musical and musical backgrounds of the respondents; the opportunities for practice, including access to space; future plans involving singing, and the place of singing in the remainder of their lifespan; messages for others (men, women, children, parents, teachers/educators, religious leaders, others) regarding singing. Also remaining for analysis are quantitative data gathered on time spent in 17 other activities such as travel, reading, clubs, choirs, orchestra/band), 12 types of entertainment (e.g., movies, radio, attending religious services, socializing). These data can contextualize further the present evidence, but they will not change the fact that voice lessons have added meaning and well-being to lives of almost all of the respondents.

### Future research

Given the evidence in this study of musical learning as well as benefits to health and well-being reported by the respondents, it would be important in future to explain what happens within that 1-hr weekly interchange of student and teacher that enables positive changes. Though motivated by the results from the present survey, such an investigation might take quite a different approach, for example, using case studies entailing video recordings of the teacher/student dyad during weekly lessons. For example, Küpers et al. (2017) studied three young string players in lessons over a period of 10 months. From multiple observers using professional coding software, quantitative measures were obtained of the autonomy of the student, autonomy support offered by the teacher, scaffolding provided by the teacher, and various student-teacher behavioral contingencies whose description is beyond the scope of the present discussion. In addition, detailed analysis of selected teacher–student verbal and gestural interactions underwent qualitative micro-analysis to reveal linkage between moment-to-moment interactions and long-term change. Comparable study of older singers using this mixed-methodology perspective would be of value from a teacher training standpoint—providing a foundation for the teaching of voice teachers of persons of all ages, sensitizing the teacher to ways in which a “high quality of scaffolding that is in tune with the student’s needs” (p. 163) can be offered, ways that may differ from those that address the younger beginner, given the life experience of the mature student, and the potentially closer match in this regard between teacher and pupil.

The present study obtained information regarding the level of musical difficulty which interested students, however, the level of performance attained from both musical and technical standpoints could be obtained in longitudinal studies across weeks, months, and years. Expert vocalists could be asked to rate the musicality, vocal beauty, creativity, or technical proficiency of performance of one piece in a controlled study, or, with less control, any piece that singers were working on. Improvement in vocal quality could be similarly assessed through analysis of the typical sustained pitches (messa di voce) of warm-up exercises that usually begin lessons. The development of vibrato could be tracked, and these measures then could be correlated with the student’s own judgments of their performance or feelings of well-being associated with voice lessons. It would be of interest to know, for example, the extent to which the sense of musical improvement or increased well-being is associated with objective measures of change in vocal skill and musical expressivity, or whether instead the value of lessons arises more from the opportunity to share music with an attentive listener, in the same way that one might benefit from time with a psychology counselor or fitness instructor.

The present study inadvertently obtained data from a sample that represented a higher than average socioeconomic status. It seems wrong that enhancement of well-being arising from voice lessons be confined to a privileged demographic. It would now be important for future experiments to determine whether voice lessons would be both feasible and beneficial for older persons of lower-than-average educational attainment and socioeconomic status. Furthermore, while the survey provided a great deal of information about the respondents, in retrospect it would have been helpful to have known the work/retirement status of the participants as this might have contextualized the availability of time to take lessons and to practice.

Of course, the cost of and time involved in taking lessons may not be feasible for those who are working two or three jobs and taking care of children or their own parents. Training from an excellent, experienced teacher, who tailors the lesson to the student (Küpers et al., 2017), may cost more than lessons from more novice teachers. Should it be found through further research that voice lessons serve therapeutic functions, for example, reducing medical problems, health care costs and increasing stability within a household, voice lessons could be a benefit that various levels of government could subsidize, being further ahead in the long run economically. Controlled experiments in which older persons from different educational and socioeconomic backgrounds are provided with the opportunity to take voice lessons should reveal the information needed.

Our evidence for the suggested relation between the age of starting voice lessons and the number of children one has begs also for further research to determine whether this effect is specific to women, who may carry the larger burden of time dedicated to child or parent care. The number of male and female participants in the present study was insufficient to examine the data further for gender differences. Future research could request that participants keep a log of their practice over the period of a week or weeks in order to obtain information regarding the relation between the amount of practice, progress, and judged benefits. The current data reporting method notwithstanding, evidence of practice durations of approximately 45 min on average are consistent with the value ascribed to voice lessons of this set of older adults.

In conclusion a survey of 48 persons, of mean age approximately 60 years, who had studied voice for approximately 5 years, since the age of 55 years, revealed evidence of the value of this activity in bringing meaning to their lives in a variety of ways that can be understood in terms of purpose, autonomy, and social affirmation (see further Creech et al., 2014), underlaid by the intrinsic value of experiencing the joy of singing challenging and beautiful repertoire. Benefits to physical health, emotional well-being, and social interactions were instantiated in different ways across the respondents. Given that the voice is an accessible musical instrument available to almost everyone, these results might motivate practitioners in the field of gerontology, for example, to consider the value of singing lessons for their clients. However, it is also important to encourage experiments that specifically compare the experience of voice lessons to lessons on another musical instrument or training of another type of activity (e.g., example theater or sport), in persons ranging in socioeconomic status, education, and ethnicity, to further clarify whether the value of voice lessons, as shown in the present study, generalizes to a broader demographic of older adults, and whether this value is unique when compared with that derived from individualized instruction in other domains.

## Supplemental Material

sj-pdf-1-pom-10.1177_03057356211030992 – Supplemental material for Singing lessons as a path to well-being in later life Click here for additional data file. Supplemental material, sj-pdf-1-pom-10.1177_03057356211030992 for Singing lessons as a path to well-being in later life by Alexandra M Smith, Kay Kleinerman and Annabel J Cohen in Psychology of Music

sj-pdf-2-pom-10.1177_03057356211030992 – Supplemental material for Singing lessons as a path to well-being in later life Click here for additional data file. Supplemental material, sj-pdf-2-pom-10.1177_03057356211030992 for Singing lessons as a path to well-being in later life by Alexandra M Smith, Kay Kleinerman and Annabel J Cohen in Psychology of Music

## Notes

1. Income estimates are rough but useful indicators. It is noted that the data were collected in 2010, when US$100 in 2010 would require US$120 dollars today for equal purchasing power. https://www.in2013dollars.com/us/inflation/2010. The same inflationary factors apply for Canadian dollars. The US and Canadian dollar were roughly at par then. The skewed socioeconomic category distribution is useful.

2. In anticipation that the richness of the data could support a variety of further investigations, an effort was made to obtain consent for anonymized data sharing; having obtained this from the majority of the respondents, the authors welcome inquiries of serious researchers who are interested in further exploration of this corpus.
