# Supplementary material for: Singing lessons as a path to well-being in later life
Source: Psychol Music. 2021 Sep 3;50(3):911–32. doi: 10.1177/03057356211030992 (PMC9014673; doi:10.1177/03057356211030992)
Supplement: sj-pdf-1-pom-10.1177_03057356211030992 – Supplemental material for Singing lessons as a path to well-being in later life [file sj-pdf-1-pom-10.1177_03057356211030992.pdf]

Questionnaire About Singing Lessons

The impact of singing lessons in later life

Thank you for responding to our invitation and consenting to complete this questionnaire. We understand that you are taking or have taken private voice lessons, and that you are willing to share some of your experiences about this. You are under no obligation to answer any or all of the questions. Please answer whatever questions you are comfortable with.

There are two parts to the questionnaire. The questions in Part I are descriptive and open-ended; the questions in Part II are short-answer about demographics and lifestyle. It may take you from 20 minutes to an hour to answer this, or perhaps longer, depending on how much you want to write. We encourage you to answer these questions as fully as you would like to.

Part I – Full sentences preferred; Point form is acceptable

1. Describe your background:
(a) non musical (for example family, childhood, adolescence, young adulthood, community, environment, opportunities, education, work/profession)
(b) musical

2. What prompted you to turn to singing?

-Wanted to since childhood

-Personal growth

-Performance goals to sing for friends

-Performance goals to sing in public

-Professional singing goals

-Singing for enjoyment

-To balance an adverse situation that was present for me when I started voice lessons

3. Describe your experiences with your teacher(s) and what was required of you in your voice lessons

4. What opportunities did you have to practice, including practicalities like space to practice?

5. What were your singing goals and did you achieve them?

a) repertoire
b) context – choral group or solo
c) performance

6a. What piece or pieces are you currently working on (composer and title if known)?
b. What is the most difficult piece, in your opinion, that you have mastered to some extent (composer
and title if known)?

7a. When you first began, describe your commitment to learning the elements of vocal technique?
b. Did that commitment change over the time during which you took lessons?

8. During the course of your lessons, describe what you learned about:

a) singing
b) yourself
c) others
d) anything else

9. Describe how singing may otherwise have impacted your life from the standpoint of:

a) physical health
b) emotional/mental health
b) personal relations
c) professional relations (e.g., leadership; interaction with colleagues and/or clients; etc.)
d) other

10. What are your future plans involving singing?

11. Looking at your future lifespan, what role do you see for singing in it?

12. Do you have a message for others in regard to singing

a) for women
b) for men
c) for children
d) for parents
e) for teachers/educators
f) for religious leaders
g) for others

13. How, if at all, did the experience of singing lessons affect your ability to express yourself in other areas?

14. Is there anything else that you would like to share about our experience with voice lessons?

Part II – Short answer

1. Gender -

2. Birth date (day/month/year i.e. 19/10/1960) -

3. Number of years of study -

4. Ethnic background –

5. Country of birth –

6. If not born in North America, at what age did you move to North America?

7. Residence:

A. Country
B. State
C. Province

8. Income level:

-Lower (0 - $20K)
-Lower Middle ($21,000 - $35K)
-Middle ($36K - $69K)
-Upper Middle ($70K – $99,000)
-Upper ($100,000 - $250,000)
-Money is no object (greater than $250,000)

9. Marital Status: Single (Never Married); Married: Cohabitating; Divorced: Widowed; Separated

10. Number of Children:

11. Lifestyle - Leisure Activities:
Please check the frequency of your participation over the past year using the following scale:
0 = Never
1 = Once per year
2 = Several times per year (up to 10 times per year)
3 = Monthly
4 = Weekly
5 = Several times per week
6 = Daily

Participating/Active in:
Sports/Athletics;
Gardening:
Choir
Dance
Visual/Fine Arts

Orchestra/Band
Acting or Performance
Social Games such as board games, card games and Chess
Cooking
Travel
Vacation Home
Visiting Family
Reading
Clubs
Crafts
Writing
Computer/Social Networking
Other (please specify)

<u>12.</u> Lifestyle - Leisure Activities:
Please check the frequency of your participation over the past year using the following scale:
0 = Never
1 = Once per year
2 = Several times per year (up to 10 times per year)
3 = Monthly
4 = Weekly
5 = Several times per week
6 = Daily

<u>Attending/Passive activities:</u>
Movies
Museums
Live Theater
Classical Concerts
Pop or other contemporary music (Rock, Folk, Hp Hop; Country and Western etc.)
TV
Radio
Continuing Education
Attending religious services; Socializing; Dining out/drinking out; Other – please specify
Socializing
Dining out/drinking out
Other _ please specify

<u>13</u>. How often do you practice?
0 = Never
1 = Once per year
2 = Several times per year (up to 10 times per year)
3 = Monthly
4 = Weekly
5= Several times per week
6 = Daily

14. When you practice how long are your practice sessions? (i.e. minutes or hours)

15. On a scale of 1 (low) to 7 (high), please rate your sense of improvement as a singer since you started voice lessons:

16. Did your voice range increase from studying voice:
1 = Not at all
2 = Somewhat
3 = A great deal

17. Did the difficulty of repertoire increase as you studied voice:
1 = Not at all
2 = Somewhat
3 = A great deal

18. On a scale of 1 (low) – 7 (high), how important to you are the following results of your singing lessons:

1. increase in voice range
2. increase in complexity of the music you are able to sing
3. increase in emotional depth of the music you are able to sing
4. increase in artistic experience
5. increased understanding of music through focused study of specific pieces
6. increased understanding of music through public performance
7. increased general sense of mastery with voice that you might
equate to say mastery of public speaking (a non-artistic aspect of competence)

19. What is your highest level of education:
a) High School ____ Bachelor Degree____ Master's Degree_____ Doctoral Degree_______
b) Please indicate the highest music credential, if any, that you have received ______________

20. How often do you take lessons?
1 = Monthly
2 = Bi-weekly
3 = Weekly

21. Is your voice teacher a member of NATS (National Association of Teachers of Singing)
a) yes b) no c) don't know
We appreciate your time and trouble. We will be pleased to provide you with a report when the data are compiled and analyzed. This will be posted on the AIRS website and we will send you an e-mail when this is available.

22. Please provide an e-mail address so that we can contact you if necessary (e.g., if a question was unintentionally left blank, or a response was unclear). You are under no obligation to provide an e-mail address.

We are very grateful for your participation in our study. Through your time today, others may benefit from your sharing of your experience of taking singing lessons.

Thank you and sincerely,

[redacted]

[redacted]
