# Supplementary material for: Singing lessons as a path to well-being in later life
Source: Psychol Music. 2021 Sep 3;50(3):911–32. doi: 10.1177/03057356211030992 (PMC9014673; doi:10.1177/03057356211030992)
Supplement: sj-pdf-2-pom-10.1177_03057356211030992 – Supplemental material for Singing lessons as a path to well-being in later life [file sj-pdf-2-pom-10.1177_03057356211030992.pdf]

Impact of Voice Lessons in Later Life

Information Letter and Consent Form

Hello,

We understand that you began taking singing lessons for the first time after the age of 40 and that you continued them for at least a year. We are interested in understanding your experiences as a later-life student of singing, and we invite you to participate in a research project that explores singing as an avenue for personal growth and wellness in later life singers. [redacted] professor of Psychology [redacted] and [redacted]) and founder of [redacted] will conduct the research. They have developed a questionnaire for this purpose. The project is part of a larger research project focusing on singing [redacted]

If you choose to take part in this project it may take you from 20 minutes to an hour of your time, or perhaps longer, depending on how long you choose to make your answers to each question. We encourage you to answer these questions as fully as you would like. No known harm will come to you if you participate in this study. Whether or not you take part is completely up to you. You may stop participating in the project at any time, without any consequences. We will keep all information that we collect during this project confidential and anonymous. We will ensure that you will not be identified from any of your responses. We will identify you only by a number or a code name in the final transcript, unless you request to be identified with your real name.

[redacted] and [redacted] are the only people who must have access to the data resulting from this research project. We will retain all of the questionnaire data for five years after the end of the project or after publication of the results, and then we will destroy it. However, the anonymous responses to questions may be made available on a web-site so that others may have access to this rich information. This would only happen if you provide permission.

If you participate in this project, you will receive no monetary or material compensation of any kind.

You may copy this consent form for your records and information. If you have any questions or concerns about this research project, please contact [redacted], or e-mail both at laterlifesinging@gmail.com

A summary of the results of the study when the data have been analyzed will be made available to you on the project web -site (http://www.airsplace.ca) and will also provide information about publications that will arise from the research. We will send an e-mail to you when the summary is available. The Research Ethics Board [redacted] has approved this research project.

What You Will Be Asked To Do

You will be asked to answer the aforementioned questionnaire in an on-line format,

with as much detail as you are willing to give. You may prepare you answers once you see the questions, and then enter them into the survey when you are ready, or you can simply respond to the questions the first time you see them. Upon completion of the questionnaire you will be asked to submit it.

Immediately after receiving the completed questionnaire, the researchers will review it to make sure there is no unclear or unintentionally missing information. If there is, the researchers will query you by e-mail. The questionnaire therefore includes a place for your e-mail address. That might compromise your anonymity. Therefore, if that is a concern for you, if your e-mail address does disclose your name or any identifying information, you are at liberty of course to create another e-mail address (e.g., in hotmail, or g-mail) that would conceal your identity. In any case you are not obliged to provide an e-mail address. Your data will never be connected with you in any reports, unless you ask us to do this (e.g., should we wish to quote something you have said). Once everything is complete, we will immediately send the following debriefing material to provide more information about the study and its implications.

Possible Benefits

There are no known direct personal benefits from participating in this study. However, the opportunity to share your story could be of benefit to you, in an altruistic sense, in that it would contribute to a burgeoning field of knowledge in which you may have interest. Also, through participation, the overall results of the study will become known, and you will learn more about the community of persons taking singing lessons in later life.

No Possible Risks and Discomforts

There are no known risks or discomforts to participating in this study.

Compensation

There is no monetary or material compensation for participating in this study.

Confidentiality and Anonymity

The data for this study will be collected on-line through *Survey Monkey,* which is set up through a confidential account. The account is password protected with the password known only to the two researchers. Therefore, data can be downloaded only by the researchers. The data will be deleted on Survey Monkey once downloaded, and the downloaded data will be retained in a locked filing cabinet in one of the investigators offices for a period of five years beyond publication date, or longer if the agency supporting the research [redacted] should require.

You as a study participant will remain anonymous (unless you specifically request to be identified in the study), since the questionnaire does not ask for your name or any identifying characteristics. Your e-mail address is requested only in the event we need to reach you for clarification or follow-up, as previously mentioned. We will not ask for a waiver of confidentiality from you.

The majority of results reported will be for group data, and will not implicate you as an individual. If your individual case is discussed in the reporting, your real name will not be used, nor will any identifying personal features or information, unless you wish to be identified, as per the consent form.

To support our discussion of the data, we may want to use direct quotes from your responses. We will contact you by your e-mail address to obtain your permission for this, unless you indicate on the consent form that we may use specific examples and/or direct quotations from your responses, without your prior consent.

For Questions About the Study, Please Contact:

[redacted] / laterlifesinging@gmail.com
Professor of Psychology at the University of Prince Edward Island and Director of the AIRS
[redacted]

[redacted] laterlifesinging@gmail.com
[redacted]

For Problems or Concerns of an Ethical Nature

If you have any difficulties with, or wish to voice concern about, any aspect of your participation in this study, or the ethical conduct of this study, you may contact [redacted] Research Ethics Board, for assistance at [redacted]

Consent Form

Title of Study: Toward A Transformative Experience with Voice Lessons in Later Life

I have read and understood the explanation about this study. I have been given the opportunity to discuss it and my questions, if any, have been answered to my satisfaction. I hereby consent to take part in this study.

I understand that:

1. My participation is voluntary and that I am free to withdraw from the study at any time.
2. I will be presented with a questionnaire about my experience taking singing lessons at or after the age of 40 years
3. I am asked to answer only the questions with which I am comfortable.
4. My privacy will be protected in that my name or contact information (other than my e-mail address) will not be requested.
5. My data will be stored in a locked filing cabinet for at least a 5 year period.
6. The data of the group of participants in this study (without any identifying features of individuals) may be made available on the AIRS research web-site so that other researchers might have access to this rich source of information, however, the inclusion of my data, would depend entirely on my permission.
7. Completing the questionnaire will take between 20 and 60 minutes (or longer if I choose)
8. I may keep a copy of this consent form, signed and dated.
9. Any ethical concerns about the study can be directed to [redacted] of the [redacted] Research Ethics Board at [redacted]

I give/do not give my consent to the following:

1) The researchers may re-contact me for possible future phases of research or other related studies: ___ Yes ____ No (If Yes, please list your contact information at the end of the survey in the space provided in Part II, item #22, and stipulate "May re-contact.")

2) I wish to be identified by name if my individual case is discussed in the reporting of the data: ___ Yes ____ No (If Yes, please list your contact information at the end of the survey in the space provided in Part II, item #22, and stipulate "May identify me by name if my individual case is discussed.")

3) Researchers may use any and all material, including specific examples and/or direct quotations from my responses, without my prior consent: ____ Yes ____ No

4) If the data from the study are made available to other researchers for additional analyses, I will allow my data to be used in view of the fact that there is no contact information or any identifying information provided about me: ___ Yes ___ No
